# The sensitivity of a honeybee colony to worker mortality depends on season and resource availability

**DOI:** 10.1186/s12862-020-01706-4

**Published:** 2020-10-29

**Authors:** Natalie J. Lemanski, Siddhant Bansal, Nina H. Fefferman

**Affiliations:** 1 Department of Ecology, Evolution, and Natural Resources, Rutgers University, New Brunswick, NJ USA; 2 Department of Ecology and Evolutionary Biology, University of Tennessee, Knoxville, TN USA; 3 Department of Ecology and Evolutionary Biology, University of California, 4114 Life Sciences Building, Los Angeles, CA 90024 USA

**Keywords:** *Apis mellifera*, Demography, Disposable soma theory, Evolution of aging, Honeybee, Life history theory, Phenotypic plasticity, Resource allocation, Senescence, Social animals

## Abstract

**Background:**

Honeybees have extraordinary phenotypic plasticity in their senescence rate, making them a fascinating model system for the evolution of aging. Seasonal variation in senescence and extrinsic mortality results in a tenfold increase in worker life expectancy in winter as compared to summer. To understand the evolution of this remarkable pattern of aging, we must understand how individual longevity scales up to effects on the entire colony. In addition, threats to the health of honey bees and other social insects are typically measured at the individual level. To predict the effects of environmental change on social insect populations, we must understand how individual effects impact colony performance. We develop a matrix model of colony demographics to ask how worker age-dependent and age-independent mortality affect colony fitness and how these effects differ by seasonal conditions.

**Results:**

We find that there are seasonal differences in honeybee colony elasticity to both senescent and extrinsic worker mortality. Colonies are most elastic to extrinsic (age-independent) nurse and forager mortality during periods of higher extrinsic mortality and resource availability but most elastic to age-dependent mortality during periods of lower extrinsic mortality and lower resource availability.

**Conclusions:**

These results suggest that seasonal changes in the strength of selection on worker senescence partly explain the observed pattern of seasonal differences in worker aging in honey bees. More broadly, these results extend our understanding of the role of extrinsic mortality in the evolution of senescence to social animals and improve our ability to model the effects of environmental change on social insect populations of economic or conservation concern.

## Background

A major challenge of life history theory is explaining the great diversity of lifespans and patterns of senescence in the natural world. Senescence, defined as a decline in physiological functioning usually accompanied by an increase in the rate of mortality with age, seems puzzling since natural selection should eliminate traits that reduce survival or fecundity.

The main evolutionary explanation of senescence is that a decline in the force of selection with age due to random mortality allows the accumulation of late-acting deleterious mutations [1, 2] or positive selection for genes that are beneficial early in life but detrimental later [3]. One physiological mechanism for this antagonistic pleiotropy is an energetic cost to somatic maintenance [4]. If there is a tradeoff between investment in reproduction and maintenance, selection may favor an optimal level of investment in maintenance that allows some accumulation of damage, resulting in senescence [4–6].

Early proponents of the above theories predicted that higher extrinsic mortality should accelerate the decline in selection with age, resulting in increased senescence [1–3]. However, further refinement has led to debate over how the force of selection changes with age and how extrinsic mortality affects the force of selection against senescence. For instance, the effect of extrinsic mortality on senescence depends on the type of density dependence [7]. In addition, the force of selection may not inevitably decline with age and can even increase [8] resulting in negligible or negative senescence [9]. These more nuanced theoretical findings may explain why there has been mixed empirical support for the prediction that higher extrinsic mortality causes faster senescence [10–16].

The honeybee (*Apis mellifera*) is a useful model system for empirically testing predictions about how changes in the force of selection influence the evolution of senescence. Honeybees have a remarkable degree of phenotypic plasticity in the rate of aging within the worker caste, with workers having up to a tenfold difference in life expectancy based on season, social environment, and task performance [17–21]. Because of their division of labor and seasonally changing environment, we would expect a large degree of variation in the selective pressure on the senescence of honeybee workers.

Although it is recognized that sociality strongly influences the evolution of senescence [22], there is a relative dearth of theory on factors affecting the force of selection against senescence in eusocial organisms. One challenge of understanding senescence in social organisms is that it can be difficult to know how changes in the longevity of individuals will scale up to effects on the whole colony, the relevant unit of selection [23]. Understanding how to estimate the selective pressure against worker senescence in honeybees can thus give us broader insights into the evolution of aging in social systems.

The question we therefore seek to address is how seasonal changes in extrinsic mortality and resource availability influence the selective pressure on worker senescence in honeybees. Using a demographic model, we ask a) how sensitive is colony growth to changes in age-dependent and age-independent worker mortality, b) how does this sensitivity differ by season, and c) do seasonal changes in the force of selection predict the observed pattern of worker senescence?

Honeybee colonies have an age-based division of labor in which young workers work inside the hive as nurses and older workers forage outside [24]. Nurses have a lower senescence rate than foragers [18] and a lower risk of accidental mortality because of the protected environment of the hive [17]. Worker lifespan also has a distinct seasonal pattern. Summer bees have the shortest lifespans of 2–6 weeks, spring and fall bees have intermediate lifespans, and winter bees have the longest lifespans of up to 20 weeks [24]. Honeybees rely on a seasonal food resource and colonies must survive the winter when they are unable to forage or rear brood. Because of seasonal changes in both extrinsic mortality and food availability, we would expect the fitness effects to the colony of changes in worker senescence to vary strongly by season. In addition, pathogens and parasites, such as *Varroa destructor*, are a significant source of overwinter mortality in honey bee colonies [25, 26]. A reduction in brood survival resulting from high parasite loads may influence the colony’s sensitivity to adult worker mortality as well.

To answer our research question, we adapt a method commonly used in demographic modeling: the Leslie matrix model. This framework is typically used to estimate the growth rate of an age-structured population and to examine how different life stages contribute to the growth of a population [27]. The relative contribution of a matrix element (i.e. age-specific survival or fecundity) to the population growth rate is called elasticity [28]. In evolutionary ecology, elasticity can be used to estimate the relative impacts of different vital rates on fitness [28]. In conservation, it can be used to determine which life stage to target to have the biggest impact on a population’s growth [27].

We adapt this method to model the growth of a social insect colony instead of a population. Since honeybee workers have little or no direct reproduction, their fitness is determined by the reproductive success of their colony [23]. We assume that the selective pressure on worker traits is proportional to the effect of the trait value on colony growth and/or survival. We therefore estimate the selective pressure on worker senescence by calculating the elasticity of colony growth to changes in worker mortality. To examine whether increased parasite loads in winter influence the selective pressures on adult worker senescence, we also repeat the elasticity analyses with a range of value for brood survival.

This method gives us a computationally simple way to estimate how different worker life stages differ in their contribution to colony growth and how changes in the vital rates of individual workers affect the fitness of the colony.

## Results

**Fig. 1:**
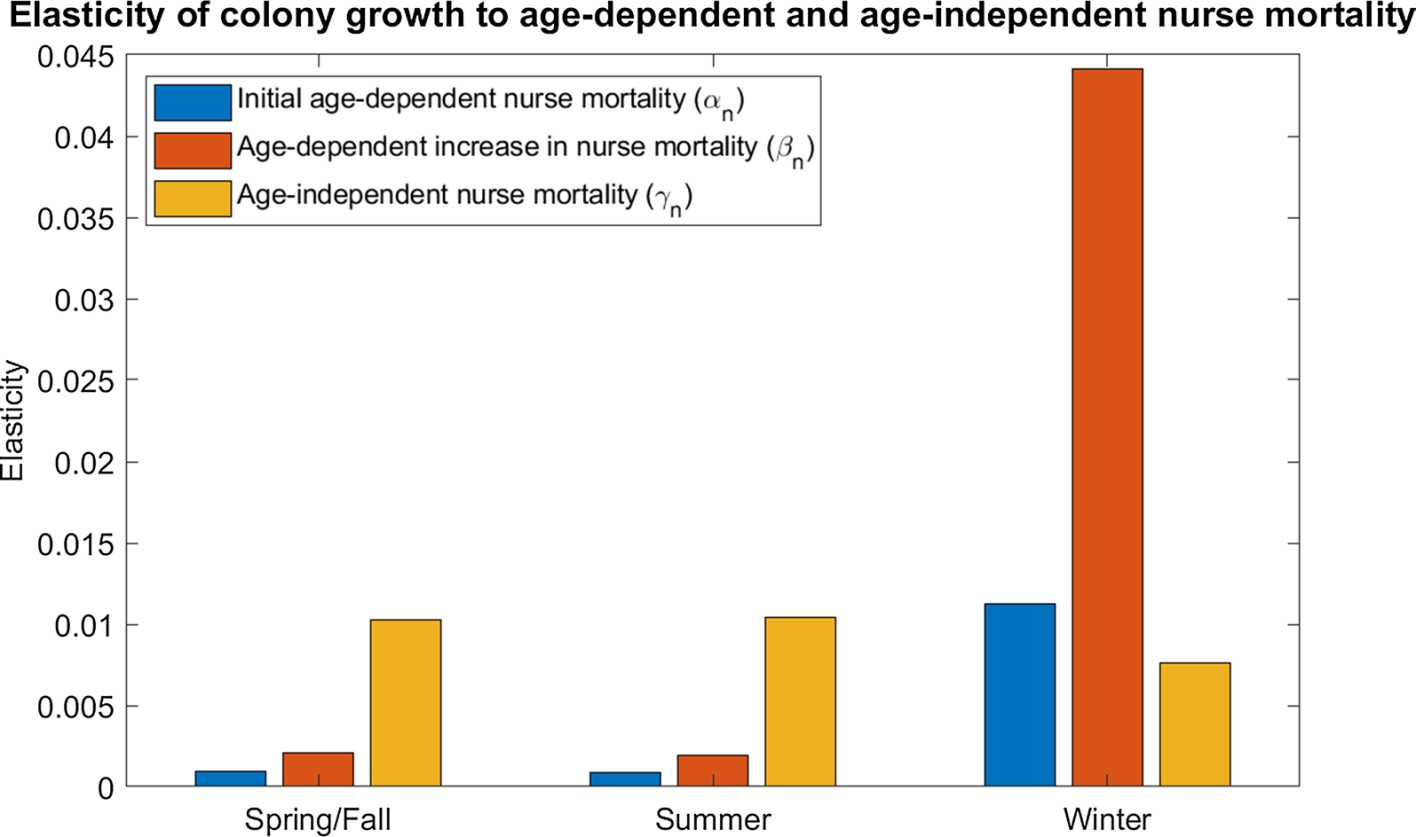
Elasticity of colony growth rate to nurse mortality. Nurse mortality is represented as a Gompertz-Makeham function, with γ_n_ as the age-independent component of nurse mortality, α_n_ as the initial age-dependent component of nurse mortality, and β_n_ as the age-dependent increase in nurse mortality. Colonies are less elastic to age-independent mortality in winter (low extrinsic mortality, low forager productivity, low forager transition rate) than in summer (high extrinsic mortality, high forager productivity, high forager transition rate) or spring (intermediate extrinsic mortality, intermediate forager productivity, high forager transition rate). In contrast, colonies are more elastic to initial age-dependent mortality and age-dependent increase in mortality in winter than in summer or spring. For full parameter values, see Additional file 1: Table S1

We find that the elasticity of the colony growth rate, λ, to the age-independent (extrinsic) component of nurse mortality, γ_n_, is highest under summer conditions (high productivity and high extrinsic mortality) and lowest under winter conditions (low productivity and low extrinsic mortality). The elasticity to γ_n_ under spring/fall conditions (intermediate productivity and extrinsic mortality) is similar to that of summer conditions (Fig. 1).

In contrast, we find that the elasticities of the colony growth rate to the age-dependent increase in nurse mortality, β_n_, and to the initial age-dependent nurse mortality, α_n_, are both highest under winter conditions and lowest under summer and spring/fall conditions (Fig. 1). Taken together, these results suggest that a honeybee colony is most sensitive to changes in nurse senescence during the winter but most sensitive to changes in nurse extrinsic mortality during the summer.

**Fig. 2:**
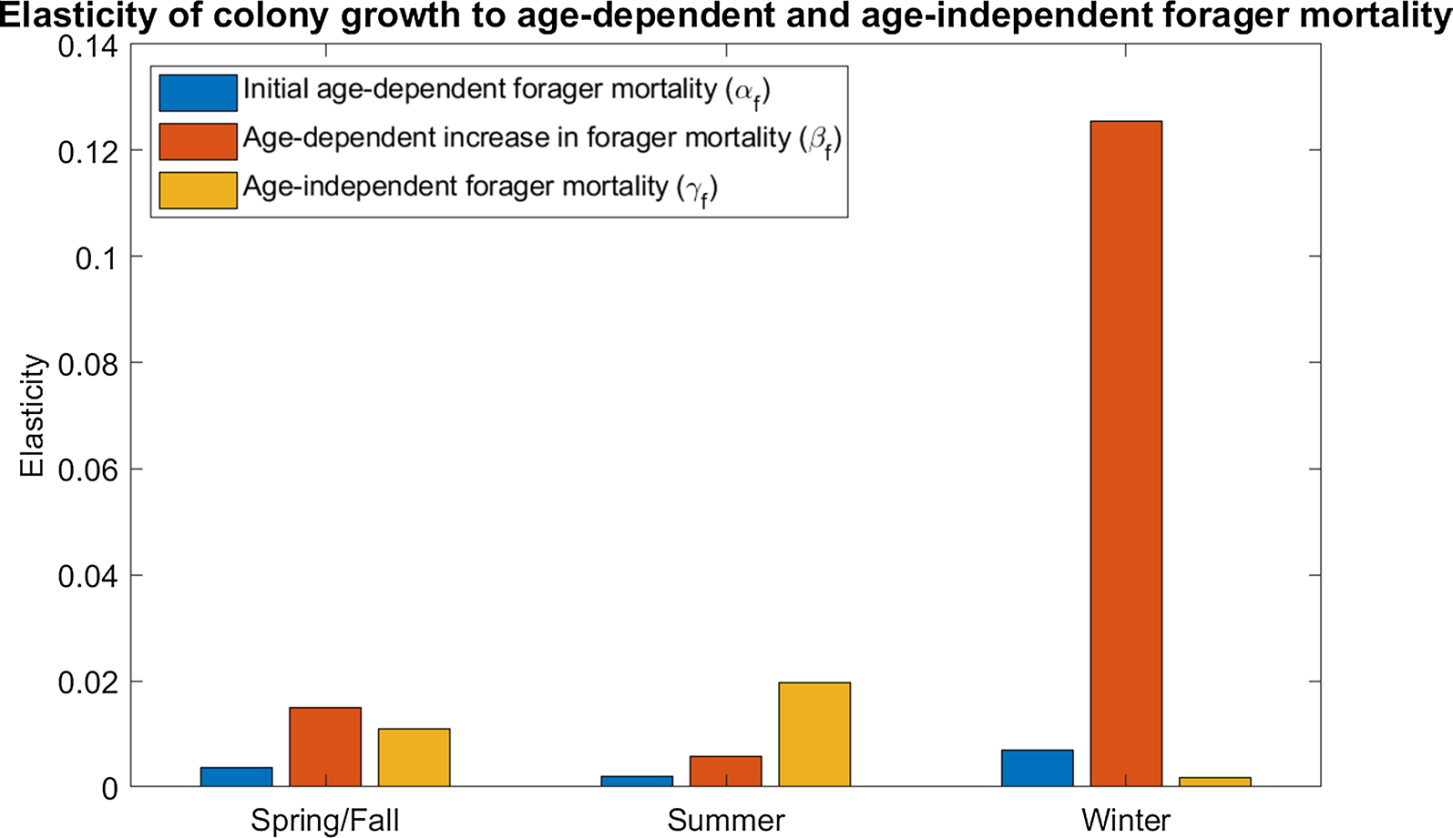
Elasticity of colony growth rate to forager mortality. Forager mortality is represented as a Gompertz-Makeham function, with γ_f_ as the age-independent component of forager mortality, α_f_ as the initial age-dependent component of forager mortality, and β_f_, the age-dependent increase in forager mortality. Colonies are least elastic to forager age-independent mortality in winter (low extrinsic mortality, low forager productivity, low forager transition rate) and most elastic in summer (high extrinsic mortality, high forager productivity, high forager transition rate), with spring/fall (intermediate extrinsic mortality, intermediate forager productivity, high forager transition rate) elasticity being intermediate between that of summer and winter. In contrast, colonies are most elastic to forager age-dependent mortality in winter and least elastic in summer, with spring/fall elasticity again being intermediate. Colonies are also most elastic to the age-dependent increase in forager mortality in winter and least elastic in summer, with spring/fall elasticity being intermediate but closer to that of summer. Note that to make seasonal differences more visible, the y-axis scale is larger in this figure than that in Fig. 1. For full parameter values, see Additional file 1: Table S1

We further find that the elasticity of the colony growth rate to γ_f_, the age-independent (extrinsic) component of forager mortality, is highest under summer conditions (high productivity and high extrinsic mortality) and lowest under winter conditions (low productivity and low extrinsic mortality). Unlike for nurse mortality, the elasticity of the growth rate to γ_f_ under fall/spring conditions is intermediate between that of summer and winter (Fig. 2). This suggests that the selective pressures against extrinsic forager mortality, like extrinsic nurse mortality, are strongest in summer and weakest in winter.

In contrast, we find that the elasticity of λ to α_f_, the initial age-dependent forager mortality, is highest under winter conditions, intermediate under spring/fall conditions, and lowest under summer conditions (Fig. 2). Similarly, we find that the elasticity of λ to β_f_, the age-dependent increase in forager mortality, is highest under winter conditions and similarly low under summer and fall/spring conditions, although it is lowest in summer conditions (Fig. 2). Together this suggests that the selective pressures against forager senescence are strongest in winter and weakest in summer.

**Fig. 3:**
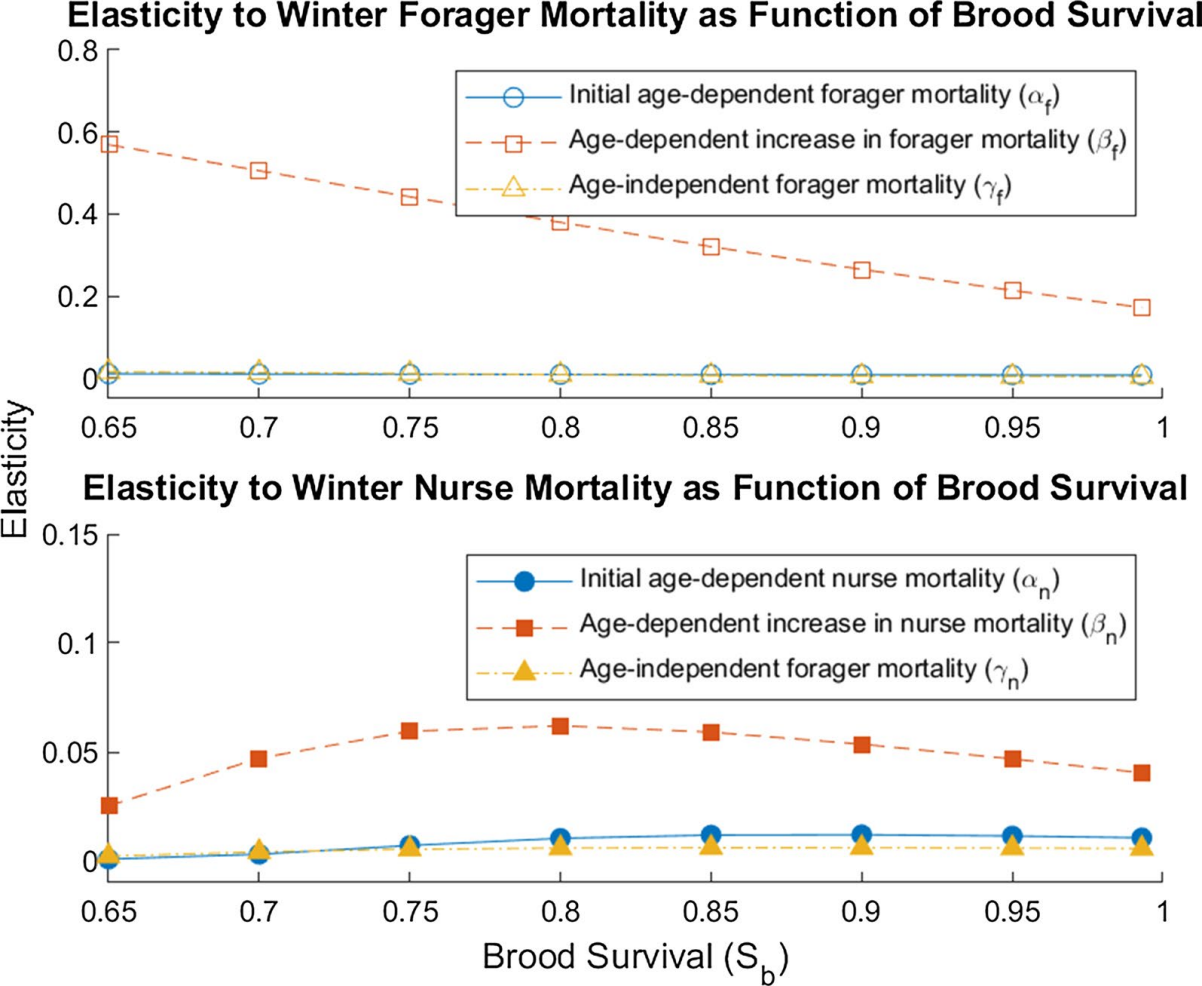
Elasticity of colony growth rate to worker mortality as a function of brood survival. To estimate how colony growth responds to changes in adult worker mortality, we calculated the elasticity for six parameters: age-independent forager mortality (γ_f_), age-dependent forager mortality (α_f_), age-dependent increase in forager mortality (β_f_), age-independent nurse mortality (γ_n_), age-dependent nurse mortality (α_n_), age-dependent increase in nurse mortality (β_n_). To examine whether reduced reduced brood survival in winter influences the colony’s sensitivity to adult worker mortality, we re-calculated the elasticities for various values of brood survival (*s*_*b*_) with all other parameters set to winter levels (Additional file 1: Table S1). Open markers show results for forager mortality parameters, while solid markers show results for nurse mortality parameters. Note that bottom panel has a smaller scale than top panel to make lines more visible

When we repeat the winter elasticity analyses with varying brood survival, we find that reduced brood survival increases the elasticity to β_f_, the age-dependent increase in forager mortality (Fig. 3). On the other hand, varying brood survival has little impact on the elasticity to forager age-independent mortality (γ_f_) or initial mortality (α_f_). Similarly, varying brood survival has very little effect on the elasticity of λ to any of the nurse mortality parameters.

## Discussion

Much of our evolutionary understanding of senescence is based on the principle that organisms experience a decline in the force of selection with age [1, 2] resulting in positive selection for traits that increase early-life survival or fecundity at the expense of late-life survival [3]. Theory further predicts that investing in somatic maintenance to postpone senescence is energetically costly [4]; when selection declines more rapidly with age, organisms should invest less in somatic maintenance and experience more rapid senescence. Differences in mean longevity and senescence rate among organisms should therefore be explained at least in part by differences in the pattern and degree to which selection changes with age.

Social insects, such as honeybees, are excellent model systems for exploring the evolution of senescence because of their large degree of phenotypic plasticity in senescence rate and lifespan among genetically similar individuals [10, 17, 29]. Different workers experience different levels of extrinsic hazards depending on their behavioral role in the colony [30]. In addition, extrinsic mortality, resource availability, and worker behavior vary seasonally, allowing us to examine how senescence in workers is influenced by ecological context.

There has been much theoretical work refining predictions about how extrinsic mortality [31], density-dependence [7], and other ecological factors [32] affect the selection against senescence in individuals. However, it is less straightforward how these ecological factors influence the strength of selection against senescence in social organisms, where individuals have little or no direct reproduction and fitness depends on their contribution to the colony as a whole. Using a simple stage-structured demographic model, we seek to bridge this theoretical gap to explore how ecological context influences selection against worker senescence in honeybees and other eusocial animals.

We find that there are seasonal differences in the strength of selection against senescence in honeybee workers, as measured by the sensitivity of the colony growth rate to age-dependent worker mortality. We find that the colony is more sensitive to changes in both nurse and forager senescence in winter conditions, when resources are scarce and extrinsic mortality is lower, than in summer conditions, when resources are plentiful and extrinsic mortality is high (Figs. 1, 2). The colony sensitivity to forager senescence is even higher when winter also reduces brood survival (Fig. 3). Since colonies cannot easily produce new workers in winter, small increases in the senescence of existing workers have larger effects on the colony. This difference in sensitivity may largely explain why winter honeybee workers have a much lower senescence rate than spring or summer workers [33]. In contrast, colonies are most sensitive to changes in extrinsic mortality (Figs. 1, 2) in summer when resources are plentiful; this may be because summer workers spend more of their lives in the riskier forager state rather than the more protected nurse state [24].

We also find the seasonal pattern of selection changes with worker life stage. There is much stronger selection against nurse senescence in winter, when most workers remain in the nurse stage, than in summer and spring/fall, both periods when they are likely to transition into foragers sooner (Fig. 1). Since nurses have much lower age-dependent and -independent mortality than foragers, selection against nurse senescence in summer is driven partly by how quickly they transition to the riskier forager state. The selection against forager senescence, on the other hand, is strongest in winter, but intermediate in spring/fall and lowest in summer (Fig. 2), suggesting that selection on forager senescence decreases as extrinsic mortality increases. This aspect of our results highlights how behavioral role can interact with ecological context to influence how the selection against senescence changes with age.

Overall, our model predicts that the selection against worker senescence should be strongest in winter and weakest in summer. This should lead to the evolution of seasonal differences in worker senescence rate, with the slowest senescence in winter and the fastest in summer. This prediction about the seasonal pattern of senescence rate matches what we observe empirically in temperate honeybee colonies [17, 33, 34]. This model therefore suggests that seasonal changes in the force of selection are important in shaping the phenotypically plastic pattern of senescence in honeybees.

Although the main objective of this model is to estimate how seasonally varying selective pressures affect the evolution of aging in honeybee workers, this method could also be used to predict how anthropogenic sources of mortality will affect the health and survival of honeybee colonies. The European honeybee is an economically important pollinator, whose crop pollination services are worth an estimated at $11.68 billion annually in the United States [35]. Managed honeybees face numerous stressors including parasites, nutrition stress, and pesticide exposure [36]. In addition, the severity of these stressors often varies by season, with the majority of colony losses usually occurring in the winter [26, 37, 38], when our model predicts that colonies are most sensitive to worker senescence. Because of logistical constraints, the impact of potential threats to honeybee health are usually evaluated at the individual rather than colony level [39]. This model can therefore help predict how changes in individual worker mortality will scale up to colony-level effects in different parts of the year, which is important to evaluating threats to honeybee health and also can give clues to the causes of colony declines [40, 41]. For instance, our model demonstrates that reduced brood survival also makes the colony more sensitive to changes in forager senescence (Fig. 3). This suggests that stressors that reduce brood survival (e.g. American Foulbrood [42], pesticide residues [43], *Varroa destructor* [26]) may act synergistically with stressors that reduce adult worker lifespan [43–45] to accelerate colony failure.

In addition, many other social insect species are of great ecological importance as pollinators [46, 47], seed dispersers [48], and ecosystem engineers [49–51]; many of these species’ populations are also threatened or declining [47, 52]. Incorporating an evolutionary perspective on how ecological context shapes resource allocation within colonies can help to better inform management practices for social species of conservation concern.

## Conclusions

The principle that selection changes with age has been a cornerstone of much of evolutionary senescence theory [1–3, 6–8]. There has been much interest in refining our understanding of how ecological factors, such as extrinsic hazards, influence the age-specific patterns of selection and in turn the evolution of lifespan. Previous work has shown that the force of selection doesn’t simply decline linearly with age, but can have more complex patterns [7–9, 53]. We here demonstrate how seasonal changes in the strength of selection can explain phenotypically plastic differences in lifespan among individuals in a social species. This simple approach to quantifying the effect of worker mortality on colony fitness can lead to better empirical predictions about how ecological factors should influence the evolution of senescence in social organisms.

### Methods

We construct an age-structured Leslie matrix model of a honeybee colony. We divide the worker population into brood, nurse, and forager stages, with each stage further divided into age classes. We define *B*_*i,t*_ as the number of *i* day old brood in the colony on day *t*, *N*_*i,t*_ as the number of *i* day old nurse bees in the colony on day *t*, and *F*_*i,t*_ as the number of *i* day old foragers in the colony on day *t*.

Rather than fecundity, as in a traditional Leslie matrix, the top row of the Leslie matrix represents the contribution of each forager to the production of new worker brood. We assume that brood development is limited only by the ability of the colony to feed them (i.e., assuming the colony is not near the queen’s egg laying capacity). We assume that workers remain in the brood stage for 21 days [24] and brood survive to the next age class with probability *s*_*b*_*.* We define *r* as the number of new brood that can be provisioned by a forager per day, with $r=\frac{p}{c}$, where *c* is the amount of food required by a brood per day and *p* is the amount of food provisioned by a forager per day. Thus, the number of *i* day old brood on day *t* is defined by:

$$B_{i,t} = \left\{ \begin{gathered} \mathop \sum \limits_{j = 1}^{19} rF_{j,t - 1} \quad i = 1 \hfill \\ s_{b} B_{i - 1,t - 1} \quad i = 2:21 \hfill \\ \end{gathered} \right.$$

We assume all adult workers start as nurse bees and become foragers after a variable number of days [54]. We define *g* as the probability a nurse becomes a forager the next day (note that this is different from a deterministic progression to forager after a fixed number of days). We assume nurse bees have a low rate of senescence and a low probability of extrinsic mortality because of the protected environment of the hive [30, 55]. We assume nurse survival is influenced by both senescence (age-dependent mortality) and extrinsic hazards (age-independent mortality) [30]. We define *s*_*n,i*_ as the daily survival probability of an *i* day old nurse and *m*_*n,i*_ as the daily mortality probability of an *i* day old nurse. We represent nurse mortality as a Gompertz-Makeham function where:

$$\begin{gathered} s_{n,i} = 1 - m_{n,i} \hfill \\ m_{n,i} = \alpha_{n} e^{{\beta_{n} i}} + \gamma_{n} \hfill \\ \end{gathered}$$

We refer to the intercept γ_n_ as the nurse extrinsic mortality parameter since it represents the age-independent component of nurse mortality. We refer to α_n_ as the initial age-dependent nurse mortality parameter and to β_n_ as the age-dependent increase in nurse mortality parameter. We assume that changes in α_n_ and β_n_ reflect changes in senescence. The number of *i* day old nurses on day *t* is given by:

$$N_{i,t} = \left\{ \begin{gathered} s_{b} B_{21,t - 1} \quad i = 1 \hfill \\ s_{n,i - 1} \left( {1 - g} \right)N_{i - 1,t - 1} \quad i = 2:120 \hfill \\ \end{gathered} \right.$$

We assume, like nurses, forager survival is influenced by both age-dependent mortality and age-independent mortality [30]. We define *s*_*f,i*_ as the daily survival probability of an *i* day old forager and *m*_*f,i*_ as the daily mortality probability of an *i* day old forager. We represent forager mortality as a Gompertz–Makeham function where:

$$\begin{gathered} s_{f,i} = 1 - m_{f,i} \hfill \\ m_{f,i} = \alpha_{f} e^{{\beta_{f} i}} + \gamma_{f} \hfill \\ \end{gathered}$$

We refer to the intercept γ_f_ as the forager extrinsic mortality parameter. We refer to α_f_ as the initial age-dependent forager mortality parameter and to β_f_ as the age-dependent increase in forager mortality parameter. As with nurses, we assume α_f_ and β_f_ represent forager senescence. We assume all workers go through a nurse stage before becoming foragers. We assume the number of workers living more than 19 days as foragers is negligible [30]. The number of *i* day old foragers on day *t* is given by:

$$F_{i,t} = \left\{ {\begin{array}{*{20}c} {\mathop \sum \limits_{j = 1}^{120} s_{n,j} gN_{j,t - 1} \quad i = 1 } \\ {s_{f,i - 1} F_{i - 1,t - 1} \quad i = 2:19} \\ \end{array} } \right.$$

To examine how the selective pressures shaping worker aging differ across annual environmental fluctuations, we modeled a colony under three different seasonal conditions: spring/fall, summer, and winter. We represented each season by different parameter values for forager extrinsic mortality (γ_f_), food availability (*p*), and nurse-to-forager transition rate (*g*). (For the full list of parameter values used in the model, see Additional file 1: Table S1) We represented summer as a season with high food availability, high extrinsic mortality, and a high nurse-to-forager transition rate. We represented fall and spring as intermediate food availability, intermediate extrinsic mortality, and a high nurse-to-forager transition rate. We represented winter as near zero food availability, low extrinsic mortality, and low nurse-to-forager transition rate since winter bees do not leave the hive to forage.

To examine the effects of forager and nurse extrinsic mortality and senescence on the growth of the colony, we performed a numeric elasticity analysis by perturbation [56]. Elasticity is a measure of sensitivity that is scaled to be unitless [27]. We calculated the elasticity of the colony growth rate (the dominant eigenvalue of the Leslie matrix) to perturbations in parameters γ_n_, α_n_, and β_n_ (the nurse mortality parameters) and γ_f_, α_f_, and β_f_ (the forager mortality parameters). If we define λ as the colony growth rate, the elasticity of the growth rate to parameter x is defined as:

$$\frac{{{\Delta }\lambda }}{{{\Delta }x}}\frac{x}{\lambda }$$

We repeated this elasticity analysis for each set of seasonal parameter conditions (Additional file 1: Table S1) to examine how the selective pressure on worker age-dependent and age-independent mortality differs by season. We assumed that brood survival did not differ seasonally. However, to examine whether reduced brood survival in winter would influence the colony’s sensitivity to adult worker mortality, we re-calculated the elasticities for various values of brood survival (*s*_*b*_) with all other parameters set to winter levels.

## Supplementary information

**Additional file 1: Table S1**. Full list of model parameters and their values.

## Data Availability

The datasets used and/or analysed during the current study are available from the corresponding author on reasonable request.
