# Supplementary material for: The sensitivity of a honeybee colony to worker mortality depends on season and resource availability
Source: BMC Evol Biol. 2020 Oct 29;20:139. doi: 10.1186/s12862-020-01706-4 (PMC7596992; doi:10.1186/s12862-020-01706-4)
Supplement: Supplementary file 1 — Additional file 1: Table S1. Full list of model parameters and their values. [file 12862_2020_1706_MOESM1_ESM.docx]

**Supplementary Information**

Table S1. Full list of model parameters and their values.

| **Parameter** | **Definition** | **Summer** | **Fall/Spring** | **Winter** |
| --- | --- | --- | --- | --- |
| *p* | Food production per forager per day | 0.098 | .058 | 0.0001 |
| *c* | Total food consumption per brood | 0.151 | 0.151 | 0.151 |
| *r* | Number of brood provisioned per forager per day | *p*/*c* | *p*/*c* | *p*/*c* |
| $s_{b}$ | Daily brood survival probability | 0.993 | 0.993 | 0.993 |
| $\alpha_{n}$ | Nurse initial age-dependent mortality rate | 0.001 | 0.001 | 0.001 |
| $\beta_{n}$ | Nurse age-dependent increase in mortality rate | 0.1 | 0.1 | 0.1 |
| $\gamma_{n}$ | Nurse age-independent mortality rate | 0.04 | 0.04 | 0.0134 |
| $\alpha_{f}$ | Forager initial age-dependent mortality rate | 0.001 | 0.001 | 0.001 |
| $\beta_{f}$ | Forager age-dependent increase in mortality rate | 0.369 | 0.369 | 0.369 |
| $\gamma_{f}$ | Forager age-independent mortality rate | 0.134 | 0.067 | 0.0134 |
| *g* | Nurse probability of becoming forager per day | 0.05 | 0.05 | 0.01 |
| *T* | Number of days considered | 90 | 90 | 90 |
